# The Role of Muscle Trigger Points in Chronic Whiplash-Associated Disorders with Neuropathic Pain Components: An Exploratory Cross-Sectional Study

**DOI:** 10.3390/jcm15093361

**Published:** 2026-04-28

**Authors:** Marta Ríos-León, Andrés Barriga-Martín, Julian Taylor

**Affiliations:** 1 Sensorimotor Function Group, Hospital Nacional de Parapléjicos (SESCAM), 45071 Toledo, Toledo, Spain; juliantaylorgreen2@gmail.com; 2 Instituto de Investigación Sanitaria de Castilla-La Mancha (IDISCAM), 45071 Toledo, Toledo, Spain; 3 School of Medicine, University of Castilla-La Mancha, 45007 Toledo, Toledo, Spain; 4 Department of Orthopaedic Surgery, Hospital Nacional de Parapléjicos (SESCAM), 45071 Toledo, Toledo, Spain; 5 Harris Manchester College, University of Oxford, Oxford OX1 3TD, UK

**Keywords:** whiplash-associated disorders, trigger points, neuropathic pain components, sensitization, disability

## Abstract

**Background/Objectives**: The role of muscle trigger points (TrPs) in neuropathic pain (NP) components in whiplash-associated disorders (WAD) has not been investigated. Our aim was to systematically investigate if referred pain elicited by trigger points (TrPs) in neck musculature reproduces neuropathic pain (NP) characteristics in chronic whiplash-associated disorders (WAD) and to determine the association of TrPs with pain intensity, mechanosensitivity, and disability. **Methods**: An exploratory cross-sectional study was conducted (n = 64; chronic WAD: n = 32; age- and sex-matched healthy controls: n = 32). TrPs in upper trapezius, suboccipital, splenius capitis, levator scapulae, scalene, and sternocleidomastoid muscles were evaluated. Pain intensity, NP components, pain catastrophizing, and disability were assessed with an 11-point numerical pain rating scale (0–10), NP questionnaires (Douleur Neuropathique 4 [DN4], self-administered Leeds Assessment of Neuropathic Symptoms and Signs [S-LANSS], and Neuropathic Pain Symptom Inventory [NSPI]), the Pain Catastrophizing Scale, and the Neck Disability Index, respectively. Mechanosensitivity (pressure pain thresholds) was assessed bilaterally over C2–C3 and C5–6 zygapophyseal joints, second metacarpal, and tibialis anterior muscle. The Mann–Whitney U test and advanced chi-square (χ^2^) test, including rank-based ANCOVA adjusted for age and sex, were used for comparisons between groups. Additionally, multivariate analyses were also performed (rank-based MANCOVA adjusted for age, sex, and pain intensity). Spearman’s rho *(r_s_)* and LOESS regression analysis, corroborated with linear regression and/or polynomial regression coefficient analysis, were used to explore associations between clinical variables in WAD. **Results**: Significant differences in distribution of TrPs, with a significant effect of sex, were found between groups (*p* < 0.05). In WAD, a greater number of active TrPs, mostly prevalent in levator scapulae and suboccipital muscles, was associated with higher pain intensity, number and intensity of NP components, and disability (0.372 < *r_s_* < 0.570, *p* < 0.05), or local mechanical hyperalgesia (*r_s_* = −0.362, *p* < 0.05). **Conclusions**: Referred pain elicited by active TrPs in the neck muscles reproduced NP symptoms in chronic WAD. This study contributes to a new understanding of pain mechanisms in WAD, highlighting the role of active TrPs in generating or maintaining NP symptoms and sensitization processes.

## 1. Introduction

Whiplash injuries are characterized by a forced flexion–extension of the neck (acceleration–deceleration), commonly after traffic crashes [1]. Whiplash-associated disorders (WAD) include symptoms/signs of pain (headache, neck pain), sensorimotor dysfunction, and psychological distress [1]. The global annual incidence of WAD following motor vehicle collisions is estimated at around 300 per 100,000 inhabitants [2,3], accounting for a greater proportion of incidence rates in females and individuals aged 20–24 years [2,4]. In Europe, a recent epidemiological study revealed an overall incidence of WAD of 267 per 100,000 person-years [2], assuming around 20.5–30% of motor vehicle collisions [5,6]. In Spain, WAD is presented in 15% of traffic accidents, leading to an estimated incidence rate of 60.2 per 100,000 person-years [7,8,9].

The most common type of WAD, according to the Quebec Task Force grading system, is WAD grade II (WAD II), characterized by neck complaints and musculoskeletal signs (e.g., muscle tenderness and decrease range of motion) without a confirmed nerve injury on routine neurological examination [10,11,12]. Nevertheless, around 34–75% of whiplash injuries present neuropathic pain (NP) components [10,13,14], with evidence of possible nervous system dysfunction [13,15,16,17,18,19,20,21,22], including WAD II, showing incomplete recovery leading to chronic pain in 50% of cases [10,12]. Thus, WAD is considered a major medical problem, with costs exceeding $230 billion per year in the United States [23,24].

The mechanisms underlying persistent pain associated with WAD and its clinical signs related to specific descriptors and sensory changes are not clearly understood in WAD [1,10,24].

Recent studies have identified putative pathophysiological mechanisms for the development of NP in WAD [10,14]. Increased T2-weighted signal intensity of spinal nerve roots [25], dorsal root ganglia [26], brachial plexus [25,26], and distal median nerve [25] apparent on magnetic resonance imaging [25,26] suggest a possible peripheral nervous system (PNS) compromise (e.g., peripheral neuroinflammation [25]) in WAD II [20,21,25,26]. Indeed, individuals with WAD II showed small-fiber structural and functional deficits, suggesting a possible pre-existing or acquired small-fiber pathology without explicit neuropathy predisposing to chronic pain [20,21,25].

Nevertheless, persistence of WAD pain may be at least partly associated with dysfunctions induced by trauma (e.g., deep neck flexor weakness and hyperactivity of superficial muscles, or forward head posture). The persistence of dysfunction, which does not resolve spontaneously even if acute pain disappears, may induce overuse of certain muscles (e.g., sternocleidomastoid, upper trapezius, and/or suboccipital muscles), leading to the potential development of TrPs and promoting a potential feedback loop for vicious circle of chronic pain until the original dysfunction is corrected [27].

Myofascial trigger points (TrPs), defined as hypersensitive spots within a taut band of skeletal muscle that are painful to mechanical stimulation and elicit a characteristic referred pain pattern [28,29], are suggested to contribute to WAD pain by acting as a peripheral source of nociception [30], facilitating central sensitization (CS) mechanisms due to an increase in nociceptive afferent input to the central nervous system (CNS) [30,31,32,33,34]. This might explain the presence of widespread pressure pain hypersensitivity (CS) in chronic WAD [30,35,36,37]. TrPs may also be developed because of secondary hyperalgesia mechanisms due to CS [32,34], mediated by neurogenic inflammation [38,39].

Previous studies have suggested a possible relationship between TrPs and NP components [40], as TrPs may be present in individuals with NP [41]. In radiculopathies, such as cervical radiculopathy [42], TrPs are more frequent (e.g., upper trapezius, splenius capitis, or levator scapulae muscles) [42], suggesting a neurogenic-like component [40]. Indeed, root compression might be considered the initiating and/or maintaining factor of active TrPs [42], linking TrPs to neurogenic mechanisms [40]. Thus, a similar potential relationship between TrPs and NP components in WAD could also be present. No study has yet investigated the role of TrPs in NP components in WAD pain.

The effectiveness of the current treatment for WAD is limited [10]; therefore, the characterization of the role of TrPs in the development or maintenance of WAD pain with NP components could help to improve its management. The aims of this study were to systematically investigate if referred pain elicited by TrPs in the neck musculature reproduced the symptoms associated with NP characteristics in individuals with chronic WAD, and to determine the association of TrPs with pain intensity, mechanosensitivity, and disability.

## 2. Materials and Methods

An exploratory cross-sectional study was conducted. The study protocol was approved by the local Clinical Research Ethics Committee and conducted according to the Declaration of Helsinki [43] and the Strengthening the Reporting of Observational Studies in Epidemiology (STROBE) guidelines [44].

### 2.1. Subjects

Participants with chronic WAD were recruited between March 2023 and January 2024 at a hospital in Toledo (Spain). All individuals screened for eligibility provided written informed consent before their inclusion in the study.

For participants to be eligible, they were required to meet the following conditions: (1) clinical diagnosis of WAD II (neck complaints and musculoskeletal signs, including decreased range of motion and point tenderness in the neck) according to the Quebec Task Force grading system [11,12,45]); (2) daily pain intensity of ≥1 rated on the 11-point numeric rating scale reported at 3 months after whiplash injury; (3) 1 or more neuropathic descriptors; and (4) adults aged 18 years or older. The exclusion criteria were (1) history of chronic pain or neurological, rheumatic, or psychiatric diseases; (2) diseases causing potential neural damage (e.g., diseases of immune system, diabetes, and oncological diseases); (3) bone injuries associated with trauma and detected in X-ray of the cervical spine; (4) previous clinical history of orofacial pain, cervical injuries (e.g., osteoarthritis, WAD, and disc herniation), or frequent headaches; (5) a history of cervical surgery or surgery to the upper extremity; and (6) treatment for chronic pain previously received over long periods of time.

Additionally, age- and sex-matched healthy controls with no history of neck pain, recruited from the general population, were also included. Exclusion criteria were the same as for the patient group.

All participants were asked to avoid any analgesic or muscle relaxant before the examination. Furthermore, an 11-point numerical pain rating scale (NPRS), ranging from 0 (no pain) to 10 (maximum pain), was used to determine the pain intensity [46] during the last 24 h (current pain intensity) and preceding week (mean pain intensity during the last week).

### 2.2. Assessment of NP Components

The presence of NP components was assessed with the Douleur Neuropathique 4 Questions questionnaire (DN4) and the self-administered Leeds Assessment of Neuropathic Symptoms and Signs (S-LANSS), as these tools showed excellent discriminant validity to detect the presence of NP components in WAD [47].

The DN4 is a reliable tool with high discriminatory value for the identification of NP components in WAD (ROC-curve value: 0.87) [47,48]. This tool includes a total of 10 items (NP descriptors), which are scored 1 for each positive (yes) item: 7 items are associated with the quality of pain (electric shocks, burning, or painful cold) and abnormal sensations (pins and needles, tingling, itching, or numbness), and 3 items are associated with clinical examination in the painful area (pinprick hypoesthesia, touch hypoesthesia, or tactile allodynia). A total DN4 score of ≥4 of 10 confirms the presence of important components related to NP [48,49].

The S-LANSS is a valid tool for identifying NP components in WAD (ROC-curve value: 0.9) [47]. The S-LANSS consists of 2 sensory examination items and 5 self-reported symptom items. Each item can be answered binarily (yes/no) (maximum sum score: 24) [47,50]. A cutoff score of ≥12 indicates the presence of NP components [50].

The Neuropathic Pain Symptom Inventory (NPSI), a valid and reliable tool for a wide range of individuals with characteristics of neuropathic pain [51,52], was also used. This questionnaire includes 10 descriptors of the different symptoms related to NP, which are scored from 0 (absence of descriptor) to 10 (maximum intensity of the descriptor). A total intensity score was calculated as the sum of the scores of the 10 descriptors. Additionally, 5 subscores corresponding to 5 subscales of this questionnaire (maximum subscore for each subscale: 10 points) are also included: burning (superficial) spontaneous pain, pressing (deep) spontaneous pain, paroxysmal pain, evoked pain, and paresthesia/dysesthesia [52].

As the DN4 and S-LANSS showed the highest sensitivity and specificity (respectively) to detect NP components in WAD [47], both tools were considered to determine the presence of important NP components in this population. Additional info related to NP components was collected using the NPSI.

### 2.3. Trigger Point Examination

An assessor with 10 years’ experience in TrP diagnosis (MRL) assessed TrPs in the upper trapezius, suboccipital, splenius capitis, levator scapulae, scalene, and sternocleidomastoid muscles according to international diagnostic criteria (i.e., presence of a sensitive spot within a taut band of skeletal muscle that elicits referred pain in response to manual compression) [28,29,30]. These muscles were chosen because their pain pattern was previously considered similar to the pain area experienced by individuals with WAD [27,34,36,53,54]. The order of evaluation was randomized between subjects, with a two-minute rest period between muscles.

A TrP was considered active if the elicited referred pain reproduced symptoms of the patient, while a TrP was considered latent if the elicited referred pain did not reproduce any symptoms considered as familiar to the patient [28]. These criteria, applied by a trained assessor, showed good inter-rater reliability (Κ: 0.64–0.88) [55], highlighting good intra-rater reliability for upper trapezius muscle [56].

In addition to the presence/absence of TrPs (active/latent), the following characteristics of pain, related to referred pain associated with active TrPs and elicited by manual stimulation of a TrP, were also considered and recorded if presented: burning pain, pressing pain, numbness, tingling, and pins and needles [28]. These sensory sensations were considered neuropathic-like symptoms related to NP components, in addition to painful cold (coldness in painful area) that is also associated with TrPs [29], as these sensations have been associated with NP components [29,47,57,58,59], particularly in WAD [47]. The frequency (number) of neuropathic-like symptoms evoked by active TrPs corresponding to the NP components reported as familiar symptoms by the patient (neuropathic-like symptoms related to active TrPs) was recorded in each muscle.

### 2.4. Mechanosensitivity

Pressure pain threshold (PPT), defined as the minimal amount of pressure where a sensation of pressure changes to pain [60], was used to assess local and widespread pressure pain sensitivity. An experienced assessor bilaterally assessed PPTs with an electronic algometer (FPIX^TM^, Wagner Instruments, Greenwich, CT, USA) in a random order at the following musculoskeletal structures: the main symptomatic areas (C2–C3 and C5–6 zygapophyseal joints [17,34,35,61,62]), one segmental-related area (second metacarpal space [34]), and a remote pain-free unrelated area (tibialis anterior muscle [17,34,35,62,63]).

Pressure was applied at a rate of approximately 30 kPa/s on each point [64,65]. The mean of 3 trials at each test point, with a resting period of 30 s between them for avoiding temporal pain summation [66], was calculated and used for the main analysis. Algometry has shown high inter-rater reliability (intraclass correlation coefficient = 0.91 [95% confidence interval, 0.82–0.97] [67], showing good-to-excellent reliability in the measurement of neck pain [63,68].

### 2.5. Pain-Related Disability and Pain Catastrophizing

Pain-related disability was assessed with the Neck Disability Index (NDI). This self-report questionnaire is a valid and reliable tool with 10 sections evaluating the severity of pain-related disability (pain intensity, personal care, lifting, reading, headaches, concentration, work, driving, sleep, and recreation), with scores ranging from 0 (no disability) to 5 (maximal disability). The maximum sum score is 50 (0–4, no disability; 5–14, mild disability; 15–24, moderate disability; 25–34, severe disability; and ≥35, complete disability) [69,70].

The Pain Catastrophizing Scale (PCS) is a 13-item self-report measure of catastrophizing in the context of actual or anticipated pain [71], and its items are scored from 0 (not at all) to 4 (all the time) on a 4-point scale [72,73,74,75]. The PCS is considered one of the most-used tools for pain catastrophizing [74,75] and measures catastrophizing as a multifaceted construct considering 3 dimensions: rumination, magnification, and helplessness [72,73,74,75]. The maximum sum score is 52, considering that higher scores indicate higher levels of pain catastrophizing [74].

### 2.6. Sample Size Considerations

Although this is an exploratory cross-section study, an estimation of sample size requirements for correlational analyses was conducted to contextualize the adequacy of the sample size. Minimum sample size was estimated using G*Power software version 3.1.9.7 (Universität Düsseldorf, Düsseldorf, Germany). The sample-size estimation was based on detecting moderate associations (r = 0.5) between the studied variables, with a two-tailed alpha level (α) of 0.05 [36] and a statistical power (β) of 80%. Under these assumptions, the estimated required sample size was calculated to be 29 individuals.

### 2.7. Statistical Analysis

Data analysis was conducted in SPSS version 23.0 (IBM Corp., Armonk, NY, USA) and Sigma Plot version 12.0 (Systat Software, Inc., San Jose, CA, USA). Results are expressed as mean, SD, or 95% confidence interval (CI). The Kolmogorov–Smirnov test revealed that data did not follow a normal distribution (*p* < 0.05); therefore, nonparametric tests were used. The Mann–Whitney U test was used for comparisons between healthy and WAD groups. The advanced chi-square (χ^2^) test was used to analyze the differences in the distribution of TrPs (active or latent) for each muscle and between groups. Additionally, the total number of TrPs was also compared between groups using non-parametric analysis of covariances (rank-based ANCOVA), adjusted for age and sex. For the WAD group, a subanalysis for differences in TrPs (dependent variables: total active TrPs and total latent TrPs) according to the presence of NP components, subdivided into two groups considering the S-LANSS (groups: S-LANSS ≥ 12; S-LANSS < 12) or DN4 (groups: DN ≥ 4; DN4 < 4) cutoff scores, was also performed. Nonparametric multivariate analyses of covariance (rank-based MANCOVA) adjusted for age, sex, and pain intensity (covariates) were included in the subanalysis. If significant multivariate effects were identified, a follow-up univariate analysis (rank-based ANCOVA) for each dependent variable was performed. Finally, Spearman’s rho *(r_s_)* and locally estimated scatterplot smoothing (LOESS) regression analysis were used to analyze the association between clinical variables and TrPs within the WAD group. Correlational analysis was also corroborated with linear regression and/or polynomial regression coefficients analysis considering LOESS plot. The statistical analysis was conducted at a 95% confidence level. A *p* value < 0.05 was considered statistically significant.

## 3. Results

### 3.1. Demographic and Clinical Data of the Participants

Thirty-nine individuals with chronic WAD were screened for eligible criteria. Seven individuals (20%) were excluded for the following reasons: previous surgery (n = 3), previous clinical history of cervical injuries (n = 2), and frequent headaches (n = 2). Finally, 32 individuals (53.1% women, mean age = 39.4 ± 11.9 years) with whiplash injury (WAD II) and 32 sex- and age-matched healthy controls (56.2% women, mean age = 36.7 ± 15.1 years) were included.

Clinical and demographic characteristics are presented in Table 1. In individuals with WAD II, the mean pain intensity (NPRS) during the last week was 5.5 ± 2.5 (Table 1), revealing significant higher mean pain intensity during the last week in women with WAD II compared to that in men with WAD II (6.1 ± 2.9 VS 3.4 ± 2.6, *p* = 0.03).

No significant differences in demographic variables existed between the groups. Significant differences in PCS were found between groups (*p* = 0.039, Table 1): individuals with WAD II presented higher scores for pain catastrophizing. Additionally, significant differences in PPTs were revealed between groups (*p* < 0.001, Table 2): individuals with WAD II showed lower PPTs than healthy controls. In fact, women with WAD II showed significant higher mechanical pain sensitivity (lower PPTs) in all points, except for C2–C3 zygapophyseal joint point, when compared with men with WAD II (Table 2, *p* < 0.05).

Regarding questionnaires for NP components, individuals with higher scores in DN4 (DN4 ≥ 4) or S-LANSS (S-LANSS ≥ 12) showed the highest mean pain intensity during the last week (DN4 ≥ 4: 7.0 ± 1.4, DN4 < 4: 3.9 ± 2.5, *p* = 0.001; S-LANSS ≥ 12: 6.0 ± 1.9, S-LANSS < 12: 3.4 ± 2.3, *p* = 0.004) and current pain intensity (DN4 ≥ 4: 6.0 ± 1.7, DN4 < 4: 2.9 ± 2.3, *p* < 0.001; S-LANSS ≥ 12: 6.1 ± 1.5, S-LANSS < 12: 3.7 ± 2.6, *p* = 0.022), levels of disability (DN4 ≥ 4: 20.6 ± 6.9, DN4 < 4: 8.6 ± 4.9, *p* < 0.001; S-LANSS *≥* 12: 20.8 ± 6.8, S-LANSS < 12: 11.8 ± 7.8, *p* = 0.002), and pain catastrophizing (DN4 ≥ 4: 19.2 ± 11.2, DN4 < 4: 8.4 ± 10.0, *p* = 0.003), in addition to lower PPTs in C5–C6 (DN4 ≥ 4: 164.2 ± 65.1, DN4 < 4: 247.1 ± 95.0, *p* = 0.015; S-LANSS ≥ 12: 134.8 ± 54.4, S-LANSS < 12: 236.5 ± 86.8, *p* = 0.003) and C2–C3 zygapophyseal joints points (DN4 ≥ 4: 151.9 ± 49.2, DN4 < 4: 200.3 ± 50.9, *p* = 0.019; S-LANSS ≥ 12: 136.6 ± 41.3, S-LANSS < 12: 193.4 ± 51.9, *p* = 0.007). No significant differences in DN4 or S-LANSS according to sex were found. Nevertheless, women showed significant higher scores in the evoked pain subscore (NPSI) compared with men (2.3 ± 1.5 vs. 0.8 ± 0.6; *p* = 0.004).

### 3.2. Distribution of TrPs and Related Neuropathic-like Symptoms in WAD

The total number of TrPs for individuals with WAD II was 4.4 ± 3.2 (4.2 ± 3.2 active TrPs and 0.1 ± 0.3 latent TrPs). The total number of TrPs for each healthy control was 0.3 ± 0.7, all of them being latent (0.2 ± 0.6). No active TrPs were observed within the healthy control group. Furthermore, the total number of TrPs was also significantly higher in individuals with WAD II than in healthy controls (*p* < 0.001). A significant effect of sex (F = 6.260, *p* = 0.015), but not age (F = 0.544, *p* = 0.464), was observed: women exhibited a higher total number of TrPs than men.

**Figure 1:**
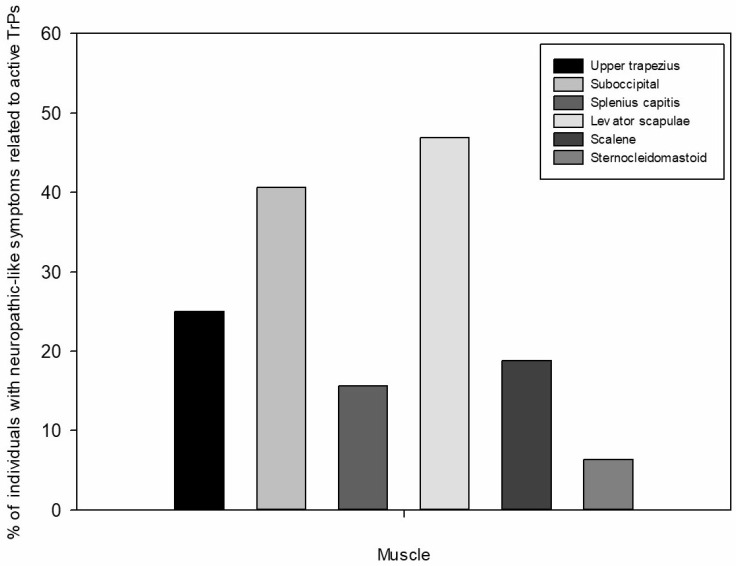
Percentage (%) of individuals with neuropathic-like symptoms related to active trigger points (TrPs) for each muscle.

The distribution of TrPs was significantly different between both groups for all muscles, except for sternocleidomastoid muscles (right upper trapezius: χ^2^ = 15.523, *p* < 0.001; left upper trapezius: χ^2^ = 11.472, *p* = 0.003; right suboccipital: χ^2^ = 31.805, *p* < 0.001; left suboccipital: χ^2^ = 29.395, *p* < 0.001; right splenius capitis: χ^2^ = 14.769, *p* < 0.001; left splenius capitis: χ^2^ = 14.588, *p* = 0.001; right levator scapulae: χ^2^ = 27.049, *p* < 0.001; left levator scapulae: χ^2^ = 21.333, *p* < 0.001; right scalene: χ^2^ = 12.528, *p* = 0.002; left scalene: χ^2^ = 8.643, *p* = 0.013; right sternocleidomastoid: χ^2^ = 3.148, *p* = 0.076; left sternocleidomastoid: χ^2^ = 2.065, *p* = 0.151). Active TrPs in the suboccipital muscles (64%), followed by levator scapulae (51.6%) muscles, were the most prevalent in individuals with WAD II, while neuropathic-like symptoms related to active TrPs were more common in levator scapulae muscles (46.9%), followed by suboccipital (40.6%) muscles (Figure 1). Table 3 presents the distribution of TrPs for all muscles in both groups.

**Figure 2:**
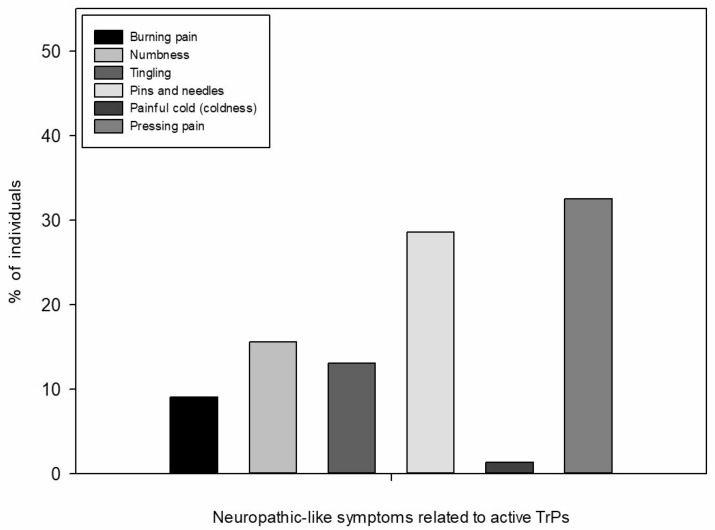
Description of neuropathic-like symptoms related to active trigger points (TrPs) and percentage (%) of individuals with each neuropathic-like symptom related to active TrPs (percentage calculated using the number of individuals with presence of each neuropathic-like symptom related to active TrPs divided by the sum of individuals with presence of any neuropathic-like symptom related to active TrPs).

Significant differences in the presence of TrPs and the presence of neuropathic characteristics were found (*p* < 0.05) according to the DN4 and S-LANSS screening questionnaires. Individuals with TrPs showed higher DN4 and S-LANSS scores compared to those without TrPs in suboccipital (DN4: 4.6 ± 1.9 vs. 2.3 ± 1.9, *p* = 0.009; S-LANSS: 11.1 ± 6.4 vs. 5.8 ± 5.4, *p* = 0.049) and levator scapulae muscles (DN4: 4.5 ± 1.8 vs. 3.1 ± 2.4, *p* = 0.034; S-LANSS: 11.9 ± 6.7 vs. 6.7 ± 5.3, *p* = 0.022). Additionally, people with DN4 ≥ 4 showed higher total number of active TrPs and total TrPs (active TrPs: DN4 ≥ 4: 5.5 ± 2.8, DN4 < 4: 3.0 ± 3.2, *p* = 0.017; total TrPs: DN4 ≥ 4: 5.6 ± 2.9, DN4 < 4: 3.2 ± 3.1, *p* = 0.017), particularly in suboccipital (active TrPs: DN4 ≥ 4: 1.7 ± 0.7, DN4 < 4: 0.8 ± 0.9, *p* = 0.023) or levator scapulae (active TrPs: DN4 ≥ 4: 1.4 ± 0.8, DN4 < 4: 0.6 ± 0.7, *p* = 0.011) muscles. People with S-LANSS ≥ 12 also presented a higher total number of TrPs in levator scapulae muscle (S-LANSS ≥ 12: 1.5 ± 0.8, S-LANSS < 12: 0.7 ± 0.7, *p* = 0.02). The most common neuropathic-like symptoms related to active TrPs were pressing pain (32.5%), and pins and needles (28.6%) (Figure 2).

Considering the subdivision on the basis of DN4 cutoff scores, rank-based MANCOVA test revealed a significant effect of current pain intensity on total active TrPs (F = 6.442, *p* = 0.02) and total latent TrPs (F = 5.972, *p* = 0.024). No significant effects of age or sex were found (*p* > 0.05). Follow-up rank-based ANCOVA confirmed significance for active TrPs (*p* < 0.05), but not for latent TrPs (*p* > 0.05).

Regarding the subdivision on the basis of S-LANSS cutoff scores, similar findings were observed. The rank-based MANCOVA test showed a significant effect of current pain intensity on total active TrPs (F = 5.131, *p* = 0.035) and total latent TrPs (F = 4.670, *p* = 0.043). No significant effects of age or sex were detected (*p* > 0.05). Follow-up rank-based ANCOVA confirmed significance for active TrPs (*p* < 0.05), but not for latent TrPs (*p* > 0.05).

### 3.3. Associations Between TrPs and Pain Intensity, Neuropathic Components, Mechanosensitivity, or Disability

Within the group of individuals with WAD II, the total number of active TrPs was significantly associated with current pain intensity (*r_s_* = 0.497, *p* < 0.001), DN4 (*r_s_* = 0.471, *p* = 0.007), S-LANSS (*r*_s_ = 0.372, *p* = 0.036), NPSI (total score: *r_s_* = 0.477, *p* = 0.006; pressing (deep) spontaneous pain subscore: *r_s_* = 0.502, *p* = 0.003; evoked pain subscore: *r_s_* = 0.570, *p* = 0.001), PPTs (C5–6 zygapophyseal joints: *r_s_*= −0.362, *p* = 0.046), and disability (NDI, *r_s_* = 0.467, *p* = 0.007) (Supplementary Table S1, Supplementary Figures S1 and S2). Table 4 shows significant correlations between frequency (number) of neuropathic-like symptoms related to active TrPs per muscle and NP components questionnaires scoring, highlighting associations between frequency of neuropathic-like symptoms related to active TrPs in levator scapulae or suboccipital muscles and S-LANSS or DN4, in addition to associations between frequency of neuropathic-like symptoms related to active TrPs in suboccipital muscles and intensity of NP components (NPSI) (Supplementary Table S2).

Additional general associations are shown in Table 5 and Table 6 (Supplementary Tables S3 and S4): greater pain intensity was associated with higher NP questionnaire scoring, higher levels of pain catastrophizing and disability, and lower PPTs (C2–C3, C5–C6); meanwhile, higher NP questionnaires scores were associated with lower PPTs (C5–C6) and higher levels of pain catastrophizing and disability.

## 4. Discussion

This study found that the referred pain elicited by active TrPs in the neck musculature may reproduce the symptoms associated with NP characteristics in individuals with chronic WAD. Additionally, having a greater number of active TrPs was associated with higher pain intensity, local pressure pain hypersensitivity (lower PPTs in the symptomatic area), and a higher number of NP components (DN4, S-LANSS) in this population. A greater number of active TrPs was also associated with higher intensity of NP components (NPSI) and higher related disability (NDI). In fact, a greater number of neuropathic-like symptoms related to active TrPs in suboccipital or levator scapulae muscles, considered the most prevalent in WAD, were associated with higher scores in NP components screening questionnaires (DN4, S-LANSS), in addition to a higher intensity of NP components (unique association with suboccipital muscle). The current results support the concept that active TrPs may be a relevant peripheral source of pain related to the extent of symptoms and suggest that active TrPs might play a role in neuropathic characteristics of pain experienced by individuals with chronic WAD.

### 4.1. Muscle Trigger Points and WAD

Similar to previous studies [27,34,36,53,54], the current research found that the referred pain elicited by active TrPs reproduces the ongoing symptomatology experienced by individuals with chronic WAD. The relationship between number of active TrPs and pain severity or pressure pain hypersensitivity in this cohort supports the importance of TrPs in pain experience and sensitization mechanisms in WAD. In fact, the number of active TrPs might influence the presence of spatial pain summation in WAD [27] related to peripheral sensitization (PS) and CS, as active TrPs, which contain high levels of algogenic substances and chemical mediators (i.e., bradykinin, substance P, serotonin, interleukin (IL)-1β, IL-6, IL-8, tumor necrosis alpha (TNF-α), norepinephrine, and calcitonin gene-related peptide (CGRP)) [76,77], could be considered relevant peripheral nociceptive inputs that trigger CS mechanisms related to the development of widespread pain and spatial pain summation [31]. Furthermore, the current study showed that a greater pain intensity was associated with a higher number of NP components (high NP questionnaires scoring) and lower PPTs, thereby supporting that higher levels and/or longer duration of pain or nociceptive input (e.g., TrPs) might influence sensitization processes and somatosensory alterations in chronic pain [78,79] in WAD explaining the presence of NP components. Previous studies have shown that a greater number of active TrPs are associated with mechanical hyperalgesia in the cervical spine (PS) and may contribute to sensitization of CNS (CS) and, hence, widespread pain sensitivity [17,34,80,81]. Furthermore, it is possible that TrPs are a consequence of CS, as CS also increases TrP sensitivity [82] potentially contributing to a vicious cycle [34,83]. Additionally, although psychological factors also might influence chronic pain in WAD [84,85,86], contradictory findings in previous studies [17,81,84,87,88], no association between pain extent and pain catastrophizing previously revealed in WAD [89], and low levels of pain-related worrying (PCS) in this sample might explain no direct significant associations with active TrPs in the current study.

### 4.2. Muscle Trigger Point-Evoked Neuropathic Pain Symptoms and WAD

This is the first study investigating the neuropathic-like symptoms elicited by active TrPs and recognized as familiar characteristics of pain experimented by individuals with chronic WAD. Previous studies have revealed a moderate presence of neuropathic-like symptoms in various musculoskeletal pain conditions [78], in addition to possible associations between duration of musculoskeletal pain and neuropathic-like pain symptoms [90]. Additionally, individuals with neuropathic-like symptoms also revealed higher pain intensity, higher levels of central sensitization-related signs and symptoms, generalized pain [91,92], and more restricted function (related disability) than those without neuropathic-like symptoms [92]. In fact, individuals with WAD exhibited higher sensitization (local and widespread pressure pain hypersensitivity) related to a greater number of active TrPs when compared with those with mechanical neck pain (local pressure pain hypersensitivity) [61,62]. Considering that a possible PNS compromise has also been identified in WAD II [20,21,25,26], the hypothesis suggesting that PNS pathophysiological mechanisms may contribute to the development or maintenance of TrPs in muscles innervated by these structures (e.g., either through a reflex mechanism or biomechanical compensations secondary to neuromuscular dysfunction) [29,40,41,42,93,94] is a possibility. Furthermore, TrPs can mimic neuropathic characteristics in other pain conditions, such as TrPs in lateral pterygoid muscle [95] or neck muscles [29,96], mimicking pain features of trigeminal neuralgia (e.g., lateral pterygoid muscle), occipital neuralgia (e.g., upper trapezius or splenius capitis muscles), or cervical radicular pain (e.g., scalene muscles) [29,95,96]. Previous studies have suggested that the presence of elevated cytokines in WAD [97,98,99], which are also present in active TrPs [76,77], may play a role in the development or maintenance of NP components [26,100]. Additionally, the high levels of pain intensity in this cohort, which correlates with the number of active TrPs, may also be associated with the presence of neuropathic-like symptoms and influence sensitization processes and somatosensory alterations [78]. In fact, the presence of neuropathic-like symptoms relates to the altered pain processing [78,79] and probably to CS present in WAD. Thus, the current findings suggest a possible relationship between TrPs and NP components in individuals with chronic WAD. Nevertheless, no causal relationship can be inferred due to the cross-sectional study design. Future longitudinal studies are now needed to confirm these findings.

### 4.3. Clinical Implications

These results support the importance of early and adequate pain management focused on identification and treatment of TrPs, which are considered at least partially responsible for the symptoms of WAD (including its possible influence on neuropathic-like symptoms). An appropriate management of TrPs may remove or decrease symptoms, including neuropathic-like symptoms, and reduce PS and CS [27], as inactivation of TrPs has been associated with attenuation of CS [33,83] and induction of spinal inhibition [39,83]. Although determining the proper treatment approach for each individual patient is challenging, a comprehensive approach, including therapeutic interventions for TrPs, may facilitate improvements in the treatment response in WAD.

### 4.4. Limitations

Some potential limitations should be considered. Firstly, the present study had a cross-sectional design; therefore, cause-and-effect relationships should not be inferred. Secondly, other musculoskeletal tissues that can also refer pain, such as joints [101], were not evaluated. Nevertheless, a careful examination of the greater occipital nerve, due to its possible entrapment related to suboccipital and upper trapezius muscles [29], or evaluation of the lower trunk of the brachial plexus, due to its possible entrapment related to scalene muscles [29], was performed to exclude individuals with confirmed nerve compression or entrapment. Additionally, an assessor with 10 years’ experience in TrPs diagnosis (MRL) assessed TrPs considering the characteristic referred pain patterns for each muscle described by Simons and Travell [29], which do not follow radicular patterns [29], thereby avoiding confounding factors related to other sources of pain. Furthermore, although potential measurement bias related to a single unblinded examiner for TrPs examination might be suggested, weak interrater reliability for TrPs classification in upper trapezius muscle has been previously reported [102]. Nevertheless, an experienced assessor provides good intra- and inter-rater reliability for TrPs diagnosis [56], which may limit potential bias. Finally, although the clinical relevance of these results should be considered with caution and might limit the generalization of these findings due to the limited sample size, this study is considered an exploratory study due to the complexity of the NP components in WAD, where underlying mechanisms remain poorly understood. Furthermore, to our knowledge, this is the first study focused on the possible role of TrPs in NP components in WAD that investigates the neuropathic-like symptoms elicited by active TrPs, exploring the possible relationship between TrPs and NP components in WAD II. Additionally, it is important to consider that the sample size was similar to or greater than that used in previous similar studies for WAD in absence of NP components [33,34]. In fact, although the sample size may be underpowered for detecting small associations, it was sufficient to detect moderate effect sizes. In addition, the effect size assumptions were based on previous studies in similar populations reporting moderate correlations between clinical variables and pain-related outcomes [36,103,104]. Nevertheless, future longitudinal studies with larger sample sizes and blinded examiners are now required to validate these associations, confirm these findings, and, hence, clarify their clinical implications.

## 5. Conclusions

Referred pain elicited by active TrPs in the neck muscles may reproduce the symptoms associated with NP characteristics in individuals with chronic WAD. Additionally, having a greater number of active TrPs was associated with higher pain intensity, higher number and intensity of NP components, local pressure pain hypersensitivity, and higher levels of disability in this population. These results support the concept that active TrPs might play an important role as peripheral pain generators in WAD, contributing to pain with neuropathic characteristics present in chronic WAD II. Nevertheless, although the present study suggests a relationship between TrPs and NP components, no causal relationship can be inferred due to the cross-sectional study design. Thus, future longitudinal studies are now needed.

## Figures and Tables

**Table 1:** Demographic and clinical variables of individuals with chronic whiplash-associated disorders (WAD) and healthy controls.

	WAD (n = 32)	Healthy Controls (n = 32)
Sex (male/female)	15 (46.9%)/17 (53.1%)	14 (43.8%)/18 (56.2%)
Age, years	39.4 ± 11.9	36.7 ± 15.1
Body mass index, kg/cm^2^	26.4 ± 5.9	24.7 ± 3.5
Duration of pain, months	3.2 ± 0.3	-
Current intensity of neck pain, NPRS (0–10)	4.5 ± 2.6	-
Mean intensity of neck pain, NPRS (0–10)	5.5 ± 2.5	-
DN4 (0–10)	3.8 ± 2.2	-
S-LANSS (0–24)	9.3 ± 6.5	-
NPSI		
Burning (superficial) spontaneous pain subscore (0–10)	1.6 ± 2.8	-
Pressing (deep) spontaneous pain subscore (0–10)	4.5 ± 3.2	-
Paroxysmal pain subscore (0–10)	2.9 ± 2.9	-
Evoked pain subscore (0–10)	2.0 ± 1.5	-
Paresthesia/Dysesthesia subscore (0–10)	3.0 ± 2.9	-
Total score (0–100)	25.3 ± 15.5	-
PCS (0–52) *	13.8 ± 11.8	7.8 ± 8.2
NDI (0–50)	14.6 ± 8.5	-

Data are presented as frequencies (n, %) or means ± standard deviation. Abbreviations: DN4, Douleur Neuropathique 4 Questions questionnaire; NDI, Neck Disability Index; NPSI, Neuropathic Pain Symptom Inventory; NRPS, numerical pain rating scale; PCS, Pain Catastrophizing Scale; S-LANSS, self-administered Leeds Assessment of Neuropathic Symptoms and Signs; WAD, whiplash-associated disorders. * Inter-group comparisons: Significant differences between individuals with WAD and healthy controls (*p* < 0.05).

**Table 2:** Pressure pain thresholds (PPTs) in individuals with chronic whiplash-associated disorders (WAD) and healthy controls.

	WAD (n = 32) ^‡^	Healthy Controls (n = 32)
C2–C3 zygapophyseal joints (kPa) *	176.9 ± 5.5 (156.7, 197.1)	313.4 ± 109.4 (273.9, 352.8)
C5–6 zygapophyseal joints (kPa) *	207.0 ± 90.9 (173.7, 240.4)	358.1 ± 115.1 (316.6, 399.6)
Second metacarpal (kPa) *		
Right side	264.7 ± 74.4 (264.7, 291.9)	365.6 ± 119.3 (322.6, 408.7)
Left side	239.2 ± 76.3 (211.2, 267.2)	349.1 ± 120.3 (305.7, 392.4)
Tibialis anterior muscle (kPa) *		
Right side	336.0 ± 102.5 (298.5, 373.6)	480.2 ± 144.4 (428.1, 532.2)
Left side	307.4 ± 122.4 (262.4, 352.3)	486.6 ± 170.7 (425.0, 548.2)

Values are mean ± standard deviation (95% confidence interval) kPa. Abbreviations: WAD, whiplash-associated disorders. ^‡^ PPTs according to sex: *C2–C3 zygapophyseal joints*: Male: 201.2 ± 55.6 (149.8, 252.6); Female: 169.8 ± 54.0 (147.0, 192.6); *p* = 0.2; *C5–6 zygapophyseal joints*: Male: 281.5 ± 108.5 (181.2, 381.9); Female: 185.3 ± 74.3 (153.9, 216.7); *p* = 0.01; *second metacarpal: right side*: Male: 333.8 ± 80.4 (259.5, 408.2); Female: 244.5 ± 60.2 (218.9, 270.0); *p* = 0.003; *second metacarpal: left side:* Male: 325.0 ± 83.0 (248.2, 401.8); Female: 214.2 ± 53.9 (191.4, 237.0); *p* < 0.001; *tibialis anterior muscle: right side:* Male: 421.8 ± 94.4 (334.5, 509.2); Female: 311.0 ± 92.0 (272.1, 349.9); *p* = 0.009; *tibialis anterior muscle: left side:* Male: 421.5 ± 165.7 (268.2, 574.7); Female: 274.1 ± 85.2 (238.1, 310.1); *p* = 0.003. * Inter-group comparisons: Significant differences between individuals with WAD and healthy controls (*p* < 0.001).

**Table 3:** Number of individuals with chronic whiplash-associated disorders (WAD) and healthy controls with trigger points in neck muscles.

		WAD (n = 32)	Healthy Controls (n = 32)
Upper trapezius (right side)	Active TrPs	12 (37.5%)	0 (0%)
	Latent TrPs	1 (3.1%)	4 (12.5%)
	No TrPs	19 (59.4%)	28 (87.5%)
Upper trapezius (left side)	Active TrPs	8 (25.0%)	0 (0%)
	Latent TrPs	0 (0%)	3 (9.4%)
	No TrPs	24 (75.0%)	29 (90.6%)
Suboccipital (right side)	Active TrPs	21 (65.6%)	0 (0%)
	Latent TrPs	0 (0%)	2 (6.2%)
	No TrPs	11 (34.4%)	30 (93.8%)
Suboccipital (left side)	Active TrPs	20 (62.5%)	0 (0%)
	Latent TrPs	0 (0%)	1 (3.1%)
	No TrPs	12 (37.5%)	31 (96.9%)
Splenius capitis (right side)	Active TrPs	12 (37.5%)	0 (0%)
	Latent TrPs	0 (0%)	0 (0%)
	No TrPs	20 (62.5%)	32 (100%)
Splenius capitis (left side)	Active TrPs	11 (34.4%)	0 (0%)
	Latent TrPs	0 (0%)	2 (6.2%)
	No TrPs	21 (65.6%)	30 (93.8%)
Levator scapulae (right side)	Active TrPs	18 (56.3%)	0 (0%)
	Latent TrPs	1 (3.1%)	3 (9.4%)
	No TrPs	13 (40.6%)	29 (90.6%)
Levator scapulae (left side)	Active TrPs	15 (46.9%)	0 (0%)
	Latent TrPs	1 (3.1%)	0 (0%)
	No TrPs	16 (50%)	32 (100%)
Scalene (right side)	Active TrPs	10 (31.2%)	0 (0%)
	Latent TrPs	0 (0%)	1 (3.1%)
	No TrPs	22 (68.8%)	31 (96.9%)
Scalene (left side)	Active TrPs	7 (21.9%)	0 (0%)
	Latent TrPs	0 (0%)	1 (3.1%)
	No TrPs	25 (78.1%)	31 (96.9%)
Sternocleidomastoid (right side)	Active TrPs	3 (9.4%)	0 (0%)
	Latent TrPs	0 (0%)	0 (0%)
	No TrPs	29 (90.6%)	32 (100%)
Sternocleidomastoid (left side)	Active TrPs	2 (6.2%)	0 (0%)
	Latent TrPs	0 (0%)	0 (0%)
	No TrPs	30 (93.8%)	32 (100%)

Data are presented as frequencies (n, %). Abbreviations: TrPs, trigger points; WAD, whiplash-associated disorder.

**Table 4:** Significant Spearman Correlation Coefficients (*r_s_*) for number of neuropathic-like symptoms related to active TrPs in neck muscles in individuals with chronic WAD.

	Number of Neuropathic-like Symptoms Related to Active TrPs in Neck Muscles
NP questionnaires	
DN4	Suboccipital muscle: *r_s_* = 0.384 (*p* = 0.03)
	Levator scapulae muscle: *r_s_* = 0.395 (*p* = 0.025)
S-LANSS	Levator scapulae muscle: *r_s_* = 0.422 (*p* = 0.016)
NPSI	
Pressing (deep) spontaneous pain subscore	Suboccipital muscle: *r_s_* = 0.397 (*p* = 0.025)
Paroxysmal pain subscore	Suboccipital muscle: *r_s_* = 0.420 (*p* = 0.017) Scalene muscle: *r_s_* = 0.435 (*p* = 0.013)
Total score	Suboccipital muscle: *r_s_* = 0.394 (*p* = 0.026)

Abbreviations: DN4, Douleur Neuropathique 4 Questions questionnaire; NP, neuropathic pain; PPTs, pressure pain thresholds; S-LANSS, self-administered Leeds Assessment of Neuropathic Symptoms and Signs; WAD, whiplash-associated disorders. These correlations were corroborated using regression analysis (see Supplementary Table S2).

**Table 5:** Significant Spearman Correlation Coefficients (*r_s_*) for mechanosensitivity, pain, or disability outcome measures in individuals with chronic WAD.

	Current Pain Intensity	Mean Pain Intensity During the Last Week
PPTs		
C2–C3 zygapophyseal joints	*r_s_* = −0.523 (*p* = 0.003)	*r_s_* = −0.474 (*p* = 0.007)
C5–6 zygapophyseal joints	*r_s_* = −0.424 (*p* = 0.018)	*r_s_* = −0.389 (*p* = 0.031)
DN4	*r_s_* = 0.689 (*p* < 0.001)	*r_s_* = 0.504 (*p* = 0.003)
S-LANSS	*r_s_* = 0.591 (*p* < 0.001)	*r_s_* = 0.551 (*p* = 0.001)
NPSI *		
Paroxysmal pain subscore	*r_s_* = 0.621 (*p* < 0.001)	*r_s_* = 0.621 (*p* < 0.001)
Evoked pain subscore	*r_s_* = 0.716 (*p* < 0.001)	*r_s_* = 0.552 (*p* = 0.001)
Total score	*r_s_* = 0.816 (*p* < 0.001)	*r_s_* = 0.630 (*p* < 0.001)
PCS	*r_s_* = 0.722 (*p* < 0.001)	*r_s_* = 0.700 (*p* < 0.001)
NDI	*r_s_* = 0.795 (*p* < 0.001)	*r_s_* = 0.640 (*p* < 0.001)

Abbreviations: DN4, Douleur Neuropathique 4 Questions questionnaire; NDI, Neck Disability Index; NPSI, Neuropathic Pain Symptom Inventory; PCS, Pain Catastrophizing Scale; PPTs, pressure pain thresholds; S-LANSS, self-administered Leeds Assessment of Neuropathic Symptoms and Signs; WAD, whiplash-associated disorder. * Current pain intensity was also associated with pressing (deep) spontaneous pain subscore (NPSI; *r_s_* = 0.632, *p* < 0.001). These correlations were corroborated using regression analysis (see Supplementary Table S3).

**Table 6:** Significant Spearman Correlation Coefficients (*r_s_*) for neuropathic characteristics, pain, or disability outcome measures in individuals with chronic WAD.

	DN4	S-LANSS
PPTs		
C2–C3 zygapophyseal joints	*r_s_* = −0.449 (*p* = 0.011)	-
C5–6 zygapophyseal joints	*r_s_* = −0.463 (*p* = 0.009)	*r_s_* = −0.533 (*p* = 0.002)
PCS	*r_s_* = 0.525 (*p* = 0.002)	*r_s_* = 0.427 (*p* = 0.015)
NDI	*r_s_* = 0.804 (*p* < 0.001)	*r_s_* = 0.663 (*p* < 0.001)

Abbreviations: DN4, Douleur Neuropathique 4 Questions questionnaire; NDI, Neck Disability Index; PCS, Pain Catastrophizing Scale; PPTs pressure pain thresholds; S-LANSS, self-administered Leeds Assessment of Neuropathic Symptoms and Signs. These correlations were corroborated using regression analysis (see Supplementary Table S4).

## Data Availability

The original contributions presented in this study are included in the article/Supplementary Materials. Further inquiries can be directed to the corresponding author(s).

## Supplementary Materials

The following supporting information can be downloaded at https://www.mdpi.com/article/10.3390/jcm15093361/s1, Figure S1: Locally estimated scatterplot smoothing (LOESS) regression (LOESS) for association between total active trigger points (TrPs) and current pain intensity (numerical pain rating scale, NPRS); Figure S2: Locally estimated scatterplot smoothing (LOESS) regression (LOESS) for association between total active trigger points (TrPs) and Neuropathic Pain Symptom Inventory (evoked pain subscore, NPSI); Table S1: Regression analysis for total number of active TrPs in neck muscles in individuals with chronic WAD; Table S2: Regression analysis for number of neuropathic-like symptoms related to active TrPs in neck muscles in individuals with chronic WAD; Table S3: Regression analysis for mechanosensitivity, pain, or disability outcome measures in individuals with chronic WAD; Table S4: Regression analysis for neuropathic characteristics, pain, or disability outcome measures in individuals with chronic WAD.
