# Supplementary material for: The Role of Muscle Trigger Points in Chronic Whiplash-Associated Disorders with Neuropathic Pain Components: An Exploratory Cross-Sectional Study
Source: J Clin Med. 2026 Apr 28;15(9):3361. doi: 10.3390/jcm15093361 (PMC13164355; doi:10.3390/jcm15093361)
Supplement: Supplementary file 1 [file jcm-15-03361-s001.zip › Ríos-León M_Supplementary Figures Caption.pdf]

SUPPLEMENTARY MATERIAL: SUPPLEMENTARY FIGURE CAPTIONS

Supplementary Figure S1. Locally estimated scatterplot smoothing (LOESS) regression (LOESS) for association between total active trigger points (TrPs) and current pain intensity. *NPRS: numerical pain rating scale.*

Supplementary Figure S2. Locally estimated scatterplot smoothing (LOESS) regression (LOESS) for association between total active trigger points (TrPs) and Neuropathic Pain Symptom Inventory (*evoked pain subscore). NPSI: Neuropathic Pain Symptom Inventory; NRS: numerical rating scale.*
