# Supplementary material for: The Role of Muscle Trigger Points in Chronic Whiplash-Associated Disorders with Neuropathic Pain Components: An Exploratory Cross-Sectional Study
Source: J Clin Med. 2026 Apr 28;15(9):3361. doi: 10.3390/jcm15093361 (PMC13164355; doi:10.3390/jcm15093361)
Supplement: Supplementary file 1 [file jcm-15-03361-s001.zip › Ríos-León M_Supplementary Tables.pdf]

Supplementary Table S1. Regression analysis for total number of active TrPs in neck muscles in individuals with chronic WAD.

	Total number of active TrPs
Current pain intensity	*Linear regression analysis*: R^2=0.264, R^2 *adj*= 0.239, *b*=0.401, *SE b*=0.122, *t*=3.278, *F*=10.742, p=0.003*
NP questionnaires	
DN4	*Polynomial regression analysis*: linear model was the unique model with statistical significance (R^2= 0.149, *F*=5.265, p=0.029).
S-LANSS	*Polynomial regression analysis:* linear model was statistically significant *(*R^2= 0.130, *F*=4.490, p=0.042) and the cubic model provided the best overall fit (R^2= 0.359, *F*=5.233, p=0.03).
NPSI	
Pressing (deep) spontaneous pain subscore	*Linear regression analysis*: R^2=0.241, R^2 *adj*= 0.215, *b*=0.480, *SE b*=0.156, *t*=3.084, *F*=9.510, p=0.004.
Evoked pain subscore	*Linear regression analysis*: R^2=0.339, R^2 *adj*= 0.317, *b*=0.260, *SE b*=0.0664, *t*=3.921, *F*=15.375, p<0.001*
Total score	*Polynomial regression analysis*: linear model was the unique model with statistical significance *(*R^2= 0.171, *F*=6.187, *p*=0.019).
PPTs	
C5-C6 zygoapophyseal joints	*Linear regression analysis*: R^2=0.131, R^2 *adj*= 0.101, *b*= - 0.986, *SE b*=0.472, *t*= -2.089, *F*=4.364, p=0.046.
NDI	*Polynomial regression analysis*: linear model was the unique model with statistical significance *(*R^2= 0.195, *F*=7.279, *p*=0.011).

Abbreviations: DN4: Douleur Neuropathique 4 Questions questionnaire; NDI: Neck Disability Index; NP: neuropathic pain; NPSI: Neuropathic Pain Symptom Inventory; PPTs: pressure pain thresholds; SE: standard error; S-LANSS: self-administered Leeds Assessment of Neuropathic Symptoms and Signs; WAD: whiplash-associated disorders.

**See Supplementary Figures 1 and 2*.

Supplementary Table S2. Regression analysis for number of neuropathic-like symptoms related to active TrPs in neck muscles in individuals with chronic WAD.

	Number of neuropathic-like symptoms related to active TrPs in neck muscles
NP questionnaires	
DN4	*Suboccipital muscle: polynomial regression analysis*: linear model was the unique model with statistical significance *(*R^2= 0.211, *F*=8.038, *p*=0.008).<br>*Levator scapulae muscle: polynomial regression analysis*: linear model was the unique model with statistical significance *(*R^2= 0.157, *F*=5.583, *p*=0.025).
S-LANSS	*Levator scapulae muscle: polynomial regression analysis*: linear model was the unique model with statistical significance *(*R^2= 0.164, *F*=5.894, *p*=0.021).
NPSI	
Pressing (deep) spontaneous pain subscore	*Suboccipital muscle*: *polynomial regression analysis*: linear model was the unique model with statistical significance *(*R^2= 0.201, *F*=7.534, *p*=0.01).
Paroxysmal pain subscore	*Suboccipital muscle:* polynomial regression analysis: linear model was the unique model with statistical significance *(*R^2= 0.159, *F*=5.657, *p*=0.024).<br>*Scalene muscle*: polynomial regression analysis: quadratic model was the unique model with statistical significance *(*R^2= 0.248, *F*=4.770, *p*=0.037).
Total score	*Suboccipital muscle: polynomial regression analysis*: linear model was the unique model with statistical significance *(*R^2= 0.235, *F*=9.215, p=0.005).

Abbreviations: DN4: Douleur Neuropathique 4 Questions questionnaire; NP: neuropathic pain; PPTs: pressure pain thresholds; SE: standard error; S-LANSS: self-administered Leeds Assessment of Neuropathic Symptoms and Signs; WAD: whiplash-associated disorders.

Supplementary Table S3. Regression analysis for mechanosensitivity, pain or disability outcome measures in individuals with chronic WAD.

	Current pain intensity	Mean pain intensity during the last week
PPTs		
C2-C3 zygapophyseal joints	*Linear regression analysis*: R^2=0.197, R^2 *adj*= 0.169, *b*= - 0.209, *SE b*=0.0784, *t*= - 2.669, *F*=7.122, p=0.012.	*Polynomial regression analysis*: linear model was the unique model with statistical significance (R^2= 0.216, *F*=7.979, p=0.008).
C5–6 zygapophyseal joints	*Linear regression analysis*: R^2=0.261, R^2 *adj*= 0.235, *b*= - 0.146, *SE b*=0.0456, *t*= - 3.199, *F*=10.237, p=0.003.	*Polynomial regression analysis*: linear model was statistically significant and selected as the preferred specification (R^2= 0.292, *F*=11.947, p=0.002), as higher-order terms did not yield significant improvements.
DN4	*Linear regression analysis*: R^2=0.452, R^2 *adj*= 0.433, *b*= 0.781, *SE b*=0.157, *t*= 4.972, *F*=24.718, p<0.001.	*Polynomial regression analysis*: linear model was the unique model with statistical significance (R^2= 0.229, *F*=8.924, p=0.006).
S-LANSS	*Linear regression analysis*: R^2=0.323, R^2 *adj*= 0.300, *b*= 0.223, *SE b*=0.0590, *t*= 3.783, *F*=14.315, p<0.001.	*Linear regression analysis*: R^2=0.243, R^2 *adj*= 0.218, *b*= 0.192, *SE b*=0.0617, *t*= 3.107, *F*=9.655, p=0.004
NPSI*		
Paroxysmal pain subscore	*Polynomial regression analysis*: linear model was the model with the best overall fit (R^2= 0.389, *F*=19.078, p<0.001).	*Linear regression analysis*: R^2=0.384, R^2 *adj*= 0.363, *b*= 0.545, *SE b*=0.126, *t*= 4.320, *F*=18.666, p<0.001).

Evoked pain subscore	*Linear regression analysis*: R^2=0.436, R^2 *adj*= 0.417, *b*= 1.155, *SE b*=0.240, *t*= 4.818, *F*=23.213, p<0.001.	*Linear regression analysis*: R^2=0.266, R^2 *adj*= 0.242, *b*= 0.893, *SE b*=0.271, *t*= 3.300, *F*=10.890, p=0.002.
Total score	*Polynomial regression analysis*: linear model was the model with the best overall fit *(*R^2= 0.662, *F*=58.646, p<0.001).	*Linear regression analysis:* R^2=0.423, R^2 *adj*= 0.403, *b*= 0.107, *SE b*=0.0228, *t*= 4.686, *F*=21.962, p<0.001.
PCS	*Polynomial regression analysis*: linear model was statistically significant *(*R^2= 0.439, *F*=23.471, p<0.001) and the quadratic model provided the best overall fit (R^2= 0.538, *F*=16.863, p<0.001).	*Linear regression analysis*: R^2=0.403, R^2 *adj*= 0.383, *b*= 0.137, *SE b*=0.0303, *t*= 4.498, *F*=20.236, p<0.001.
NDI	*Polynomial regression analysis:* linear model was statistically significant and selected as the preferred specification *(*R^2= 0.631, *F*=51.228, p<0.001), as higher-order terms did not yield significant improvements.	*Linear regression analysis:* R^2=0.405, R^2 *adj*= 0.385, *b*= 0.190, *SE b*=0.0420, *t*= 4.515, *F*=20.385, p<0.001.

Abbreviations: DN4: Douleur Neuropathique 4 Questions questionnaire; NDI: Neck Disability Index; NPSI: Neuropathic Pain Symptom Inventory; PCS: Pain Catastrophizing Scale; PPTs: pressure pain thresholds; SE: standard error; S-LANSS: self-administered Leeds Assessment of Neuropathic Symptoms and Signs; WAD: whiplash-associated disorders.

*Current pain intensity was also associated with pressing (deep) spontaneous pain subscore (NPSI; polynomial regression analysis: linear model was statistically significant and selected as the preferred specification *(*R^2= 0.373, *F*=17.854, p<0.001), as higher-order terms did not yield significant improvements).

Supplementary Table S4. Regression analysis for neuropathic characteristics, pain or disability outcome measures in individuals with chronic WAD.

	DN4	S-LANSS
PPTs		
C2-C3 zygapophyseal joints	*Linear regression analysis:* R^2=0.195, R^2 *adj*= 0.167, *b*= - 0.180, *SE b*=0.0680, *t*= - 2.651, *F*=7.026, p=0.013.	*Not performed as no significant correlation* (r_s) *was found.*
C5–6 zygapophyseal joints	*Linear regression analysis:* R^2=0.211, R^2 *adj*= 0.184, *b*= - 0.113, *SE b*=0.0408, *t*= - 2.783, *F*=7.744, p=0.009.	*Linear regression analysis:* R^2=0.249, R^2 *adj*= 0.223, *b*= - 0.358, *SE b*=0.116, *t*= - 3.102, *F*=9.623, p=0.004.
PCS	*Linear regression analysis*: R^2=0.204, R^2 *adj*= 0.177, *b*= 0.0845, *SE b*=0.0305, *t*= 2.772, *F*=7.686, p=0.009.	*Polynomial regression analysis:* quadratic model was the unique with statistical significance *(*R^2= 0.227, *F*=4.266, p=0.048
NDI	*Linear regression analysis*: R^2=0.557, R^2 *adj*= 0.543, *b*= 0.194, *SE b*=0.0315, *t*= 6.145, *F*=37.761, p<0.001.	*Linear regression analysis:* R^2=0.298, R^2 *adj*= 0.275, *b*= 0.419, *SE b*=0.117, *t*= 3.573, *F*=12.765, p=0.001.

Abbreviations: DN4: Douleur Neuropathique 4 Questions questionnaire; NDI: Neck Disability Index; PCS: Pain Catastrophizing Scale; PPTs: pressure pain thresholds; SE: standard error; S-LANSS: self-administered Leeds Assessment of Neuropathic Symptoms and Signs.
